# Monthly Summary

**Published:** 1896-04

**Authors:** 


### Monthly Summary.

BICYCLING FOR WOMEN.—A local preacher who enjoys some notoriety as a sensationalist and a *poseur* has taken it upon himself, with all the callow rhetoric of a country schoolmaster, to denounce the bicycle as a most diabolical invention, especially designed and executed for the subversion of the morals of women. In the face of such anathemas hurled from the very horns of the alter, she must be a brave woman indeed who would continue in the error of her way.

If the fervid eloquence of the very reverend doctor had been tempered with cold facts, the way of the gentle transgressor would not have seemed so hard. These facts, so conspicuously absent in the clerical denunciation of the bicycle, are well set forth in our ever dignified and reliable contemporary, the Chicago *Medical Standard,* and apply both to the male and female offenders. In a recent editorial on this subject it says: "A very severe indictment can be brought against bicycle abuse, but these abuses are common to a hundred methods of outdoor exercise. There is no necessary emotional excitement about cycling. Despite its widespread use, racing is rarer with the wheel than with the horse. 'Scorching' has been largely, if not entirely, responsible for all evils charged to the wheel. Wheel kyphosis is a result of bad position and, as women cyclers have shown, avoidable. A very calm, judicial critic, Dr. Justus Champoniere, declares that for grace and regularity of motion woman is man's superior in the use of the bicycle. She cycles as well as she dances.

"To the sedentary clerk the wheel has brought fresh air and exercise previously impossible from the cost of horses. By hygienic dress the wheel has benefitted woman to an extent which far outweighs any possible evils resultant on its use. It has doomed the corset and waist-attached skirts, the source of unlimited pelvic evils The ease and freedom with which outdoor exercise is obtained has tempted into the sunshine women with moping tendencies to invalidism. To none is it likely to prove a greater boon than to the farmer woman. Among these, because of isolation, nervous disease and insanity are rife. Decades ago Dr. Patterson, who had studied insanity in Iowa, Ohio, Indiana and Illinois farming regions, remarked: 'The wives and daughters of farmers during inclement seasons have fewer comforts connected with outdoor life and less adequate protection from cold and humid air than the women who live in our towns and cities.' It is probable, taking prairie farm life with all its surroundings, that the average standard of vital force in those who live upon farms is below that of those who live in cities and towns. It must not be inferred, however, from these suggestions that the noble and pleasing pursuits of agriculture favor the production of insanity. The errors of living and the discomforts alluded to are not necessarily connected with, and certainly not limited to farm life. Precedent to insanity always occur malaise and auto-intoxication, with resultant hepatic, renal and gastro intestinal disorders and consequent cardiac stress. Much of the so-called dyspepsia of farmers is gastro-neurasthenia. Open-air exercise (not athletics) and visiting, which the bicycle can secure, break up the dead routine underlying invalidism. Bicycling, like all exercise, has its dangers, but it has become of such practical use that there are less proportionately than those of other modes. To point out abuses, is to prevent them."

The most important objection to bloomers is their unsightliness. No woman can afford to make herself ridiculous, and with the use of bloomers, they come perilously near it. There can be no doubt but that this garb is merely transitional, and is designed as a compromise measure before the introduction of the more sensible knickerbockers.

*Mens sana in corpore sano.* Let the women ride.—*Southern Medical Rrcord.*

---

MEDICAL AND DENTAL STATE BOARDS.—The present Medical Board of Health of Missouri has recently showed commendable activity in the supervision of requirements for admission of students to colleges whose diplomas are to be accepted and registered. In the matter of preliminary requirements for freshmen, the following is one of the rules: "The candidate must have a diploma of graduation from a good literary or scientific college or high school, or a first-class teachers' certificate." In carrying out the aforesaid requirement, colleges were required to furnish the board with the names, addresses and qualifications of those admitted according to the rules. One prominent college in the State refused to obey, and claimed the rule to be arbitrary. It was accordingly boycotted by the board, and then the trouble began. The boycotted college tried to prejudice the public through the daily press, with more or less success. The board, however, "stood pat," and the result is that the college has swung into line with the board's requirements, and the boycott is removed. This result leads us to speak of the benefit resulting from a State board, where there exists a law regulating the practice of medicine or dentistry. In Missouri the enforcement of the dental law—in the absence of a board for that purpose—has been accomplished by the Missouri State Dental Association through its law committee, and, so far as the power of that committee extended, the work has been very well done, and the proffession is indebted to Dr. Jas. A. Price, the committeeman. So long, however, as the power of registration to practice dentistry in the State belongs to the county clerk, he cannot always discriminate as to the "reputability" of diplomas. Under the laws of Missouri, a charter for a dental college can be secured for a small fee by any five men, and, be their standing in dentistry ever so indifferent, their diplomas may often be credited and registered by the county clerk for the only reason that the institution holds a charter from the Missouri Assembly, and the county clerk has, in many instances, no means of knowing whether the school issuing the diploma is reputable or not. Under the circumstances, the profession in Missouri should ask the Legislature to amend the dental practice act, providing for a board whose duty it shall be to pass upon candidates for registration, and to look after the enforcement of the law, punish violators, etc.

Until recently we have believed it to be the part of wisdom to allow the law to remain as it stands, but so long as Tom, Dick and Harry can secure a charter, and county clerks are prone to consider anything chartered legitimate, it is time that the respectable men in dentistry in Missouri insist upon an examining board. It is, we believe, the one thing that should be done by the profession in Missouri during the coming year The enforcement of the law must no longer be left to private efforts, but must be done by properly constituted State authority. The recent success of the Medical Board of Health should encourage the belief that a dental board will also be granted by the Legislature, and that the public will appreciate its legitimate work.—Editorial in *Western Dental Journal*.

---

HEMORRHAGE.—Otto Arnold, D. D. S., of Columbus, Ohio, in a paper entitled "Alveolar Dental Hemorrhages," read before the Tri-State dental meeting, says:

My universal method of procedure in the management of hemorrhage following tooth extraction, is to have the patient rinse the mouth freely with hot water. This encourages free and uninterrupted bleeding from the wound, and stimulates a normal reaction in the tissues, soon followed by a natural cessation of the hemorrhage.

If any considerable amount of laceration has taken place, I attempt to replace the tissues by compressing with the fingers, or stitching into position any pendant of the gum. prescribing as a dressing:

| | | |
|---|---|---|
| R | Tannic acid, - - - - | gr. xx. |
| | Listerine, - - - - | |
| | Aqua Dest. *aa*, - - - - . | ℥ iv. |

M. Sig.—Apply frequently to the wound.

---

HYPNOTISM IN SURGERY.—Fifty years ago Dr. L. A. Dugas, of Georgia, operated three times upon a woman in the hypnotic state for recurring carcinomena of the breast. In the third operation the axillary glands were also removed. The patient gave no indication whatever of sensibility (*Southern Medical and Surgical Journal*, March, May and September, 1845). Dr. Dugas believed that the phenomena of "mesmeric sleep" might be so utilized as to "render them available to all sufferers." A little earlier Dr. Crawford W. Long, the discoverer of anesthesia, had investigated the subject and disused hypnotism because of its uncertainty of action Hypnotism has thrice been revived and twice fallen into disuse during the last century.—*Medical Standard*.

New Method of General Narcosis.—Rosenberg, in the Berlin Medical Society (Deutsche medicinische Wochenschrift, No. 50, 1894), describes a new method for including general narcosis. Before commencing the anesthetic, the patient's nasal mucous membrane is to be sprayed with a ten per cent. solution of cocaine. During prolonged anesthesia, the spraying is to be repeated every thirty minutes, and again at the end of the operation, no matter how short. His conclusions are. (1) excluding cases of negligence and of poisoning, cardiac syncope in chloroform narcosis is, in so far as it can be attributed to the chloroform, reflex. (2) The syncope and the accompanying respiratory embarrassment are due to irritation of the peripheral trigeminal filaments in the nasal mucous membrane. (3) Every anesthetic inhaled produces the same reflex symptoms as chloroform. (4) By proper cocainization of the nasal mucous membrane, all such reflexes can be positively prevented. (5) Thus a greater part of the dangers of inhalation anesthesia, particularly of chloroform, can be removed. (6) Chloroform possesses a certain action antidotal or antitoxic to chloroform, and thus further reduces the dangers of chloroform. (7) Chloroform in such cases is to be preferred to ether, as being less dangerous. The chloroform must always be administered drop by drop.—"Universal Medical Magazine."

---

Air-Chambers.—If the always successful insertion of full upper plates in all materials, rubber, gold, aluminum, continuous, gum, without air-chambers, lines made in the plaster cast, or other devices, does not demonstrate their usefulness, what further evidence is needed?

As a rule, it is rarely ever necessary to make any other change in the plaster model than a slight relief over the hard palate, so the plate shall not rock, as that is the only portion of the upper jaw which never changes nor yields to pressure. The plate will adhere just as well without this "relief."

I have on my shelves hundreds of models of every conceivable shape and condition of jaws, upon which metal plates have thus been made successfully.—L. P. Haskell in *Dental Cosmos.*

"DOC."—If it has been your misfortune to be called "doc" and if this recognition has become at all general among your friends you might as well move to some other place. A man may be called a thief, a liar and a dead beat, and yet he may prosper and live upon the fat of the land. But once let him be called "doc" and his professional success is at an end. We would prefer to spend a night in the station-house, so far as its effect on our professional success is concerned rather than to have our friends notice our approach by saying, "There comes doc." If a man calls you "doc" you need never expect a penny from him for any professional service you could render. His answer is sure to be, "All right, doc, in a few days that will be all right. "Doc" means disaster. "Doc" is the culmination of all calamity. "Doc" is a catastrophe given at one stroke. "Doc" is the warning that we have reached the extreme limit of our usefulness. "Doc" is the hand which points us to the next town. Shun it, my young friend, as you would flee from a Kansas cyclone or a praririe-fire. Kn[illegible]ck the man down who dares to speak it to you; and call upon the whole medical profession for vindication of your righteous deed.—*The National Medical Review.*

---

AS AN OBTUNDANT.—Dr. F. F. Van Woert, of Brooklyn, uses a 50 per cent. solution of sulphuric acid as an obtundant for sensitive dentine. He discovered it by accident, while endeavoring to open the pulp canals of a devitalized tooth with the acid solution. The rubber dam was over the dead tooth, and also over a carious bicuspid which stood next it. In some way the solution got into the cavity of the living tooth, which he had in vain been striving to excavate. To his surprise, he found that the tooth which had been agonizingly sensitive, was now completely obtunded. Since that time he has been using it with very gratifying results, although not in every case with complete success. Usually, however, it enables him to excavate painlessly. He uses but little at a time, and as the affinity is soon satisfied it becomes self-limiting and will do no harm. If too much is used, it can be readily neutralized with a solution of soda bicarbonate.—"Dental Pract. and Advertiser.

